# HDAC6-specific inhibitor suppresses Th17 cell function via the HIF-1α pathway in acute lung allograft rejection in mice

**DOI:** 10.7150/thno.44961

**Published:** 2020-05-21

**Authors:** Wenyong Zhou, Jun Yang, Gaowa Saren, Heng Zhao, Kejian Cao, Shijie Fu, Xufeng Pan, Huijun Zhang, An Wang, Xiaofeng Chen

**Affiliations:** 1 Department of Thoracic Surgery, Shanghai Chest Hospital, Shanghai Jiao Tong University, Shanghai, China.; 2 Department of Intensive Care, Huashan Hospital, Fudan University, Shanghai, China.; 3 Department of Cardiothoracic Surgery, Huashan Hospital, Fudan University, Shanghai, China.

**Keywords:** lung transplantation, acute rejection, mouse, HIF-1α, Th17 cells, HDAC6i

## Abstract

**Background:** Previous animal experiments and clinical studies indicated the critical role of Th17 cells in lung transplant rejection. Therefore, the downregulation of Th17 cell function in lung transplant recipients is of great interest. **Methods:** We established an orthotopic mouse lung transplantation model to investigate the role of histone deacetylase 6-specific inhibitor (HDAC6i), Tubastatin A, in the suppression of Th17 cells and attenuation of pathologic lesions in lung allografts. Moreover, mechanism studies were conducted *in vitro*. **Results:** Tubastatin A downregulated Th17 cell function in acute lung allograft rejection, prolonged the survival of lung allografts, and attenuated acute rejection by suppressing Th17 cell accumulation. Consistently, exogenous IL-17A supplementation eliminated the protective effect of Tubastatin A. Also, hypoxia-inducible factor-1α (HIF-1α) was overexpressed in a lung transplantation mouse model. HIF-1α deficiency suppressed Th17 cell function and attenuated lung allograft rejection by downregulating retinoic acid-related orphan receptor γt (ROR γt) expression. We showed that HDAC6i downregulated HIF-1α transcriptional activity under Th17-skewing conditions *in vitro* and promoted HIF-1α protein degradation in lung allografts. HDAC6i did not affect the suppression of HIF-1α^-/-^ naïve CD4^+^ T cell differentiation into Th17 cell and attenuation of acute lung allograft rejection in HIF-1α-deficient recipient mice. **Conclusion:** These findings suggest that Tubastatin A downregulates Th17 cell function and suppresses acute lung allograft rejection, at least partially, via the HIF-1α/ RORγt pathway.

## Introduction

Lung transplantation is the final therapeutic option for select patients with a variety of end-stage pulmonary diseases. However, acute rejection of lung transplantation is an independent risk factor for chronic lung allograft dysfunction (CLAD) 1,2. Despite the wide application of immunosuppression strategies, at least one episode of acute rejection occurs in one-third of adult lung transplant patients within a one-year follow-up 3. Recent reports have implicated Th17 cells and proinflammatory cytokine IL-17 (IL-17A) not only in acute lung allograft rejection but also in CLAD after lung transplantation in humans and animals 4-7. In a previous study 8, we found that the adoptive transfer of induced regulatory T cells (iTregs) downregulated Th17 and IL-17^+^ γδ T cells in the allograft recipients and attenuated the pathology of acute lung allograft rejection. However, exogenous iTregs are at risk of losing the forkhead box P3 (FoxP3) phenotype and converting into Th17 cells in an inflammatory environment. Most upregulated Th17 cells are derived from other T cells (naïve T cells, Treg cells, etc.) in the inflammatory environment 9-13. Therefore, it is important to find different ways to inhibit the function of Th17 cells in lung transplantation.

Histone deacetylase (HDAC) family is classified structurally into class I (HDAC1, HDAC2, HDAC3, HDAC8), class IIa (HDAC4, HDAC5, HDAC7, HDAC9), class IIb (HDAC6, HDAC10), class III (SIRT1-7), and class IV (HDAC11) groups 14. Class I HDAC enzymes are expressed in all cells and exhibit deacetylase activity, whereas class II HDAC enzymes have tissue-specific expression and are enzymatically inactive and act primarily as scaffolding or recruiting proteins within large multimolecular complexes 15. HDAC plays a role in the deacetylation of non-histones and regulates the biological activity of proteins through non-deacetylation 16-20. HDAC inhibitors were initially developed as anti-cancer agents, but since then, clinical and molecular data have been accrued related to their effects in anti-inflammation 21-23. Some specific HDAC inhibitors have shown the potential to alleviate the rejection of transplanted organs 24-27.

TGF-β, IL-6, IL-21, and IL-23 cytokine signals play a critical role in Th17 cell differentiation from naïve CD4^+^ T cells upon stimulation by the antigen 28-30. Retinoic acid-related orphan receptor γt (RORγt) is the key transcription factor that orchestrates the differentiation of Th17 cells 31-33. RORγt induces transcription of the genes encoding IL-17A and IL-17F in naïve CD4^+^ T cells and is required for their expression in response to IL-6 and TGF-β 34-36. Broad-spectrum or specific HDAC inhibitors suppress the expression of RORγt and inhibit the differentiation of Th17 cells* in vitro* and *in vivo* 37-46. It remains to be determined whether HDAC inhibition could attenuate lung allograft rejection by downregulating the function of Th17 cells. Moreover, because HDAC family members are involved in complex signaling networks in a variety of cells 18,20,47-49, it is essential to determine the mechanism by which HDACs independently affect Th17 cell function in lung allograft transplantation and ascertain which specific HDACi would be useful. We hypothesized that HDAC-specific inhibitors could affect graft outcomes after lung transplantation 25-27,50-53.

Hypoxia-inducible factor-1α (HIF-1α), the main functional subunit of HIF-1, mediates the transcriptional regulation of cellular and developmental response to hypoxia. The function of h HIF-1α is mainly regulated by O_2_-dependent pathways 54. Recent studies found that HDAC activity triggered HIF-1α degradation and repressed HIF-1α function 55-60. Pan-HDAC inhibitors, including Trichostatin A (TSA) and suberoylanilide hydroxamic acid (SAHA), induce O_2_-independent destabilization of HIF-1α 59,61. Furthermore, HIF-1α was shown to regulate Th17 cell fate determination 62-65. Recent studies showed that HIF-1α enhanced the development of Th17 cells from naïve CD4^+^ T cells via direct transcriptional activation of ROR γt in normoxic and hypoxic micro-environments 66-69. HIF-1α-deficient T cells were resistant to transformation into Th17 cells both *in vivo* and* in vitro* 63. Other studies revealed that the HIF-1α-dependent glycolytic pathway orchestrated a metabolic checkpoint for the differentiation of Th17 cells 64. These findings highlight the potential role of HIF-1α as a bridge to link HDACs function and Th17 cell signaling.

In the present study, we showed that HDAC6, a Class IIb HDAC, was abnormally expressed in acute lung allograft rejection in mouse orthotopic lung transplantation models. Herein, the HDAC6-specific inhibitor (HDAC6i) Tubastatin A was used to block HDAC6 in lung allograft transplantation in mice and Th17 cell differentiation *in vitro*. We demonstrated that HDAC6 inhibition downregulated HIF-1α activity and protein expression; it also strikingly attenuated the progression of acute lung allograft rejection by decreasing the function of Th17 cells.

## Materials and Methods

### Animals

Specific pathogen-free male mice (C57BL/6^H-2b^, BALB/c^H-2d^) were purchased from Shanghai Laboratory Animal Company (Shanghai, China). C57BL/6-HIF-1α^-/-^ mice were bred and maintained in the animal facilities of Shanghai Chest Hospital, Shanghai Jiao Tong University, under specific pathogen-free conditions. According to our previous experience, the larger bodyweight of recipient mice was beneficial to the survival rate and postoperative tolerance of lung transplantation. Male mice weighed more than female mice at the same age. Therefore, male mice aged 10 to 12 w were used as donors and recipients. All experiments conducted under a protocol approved by the Animal Care and Use Committee of Shanghai Jiao Tong University.

### Establishment of orthotopic mouse lung transplantation models

We have described the orthotopic mouse lung transplantation procedure in our previous study 8. Briefly, to simulate acute rejection after lung transplantation in humans, we established an orthotopic mouse lung transplantation models involving major histocompatibility antigen (MHC)-mismatched transplantation models (BALB/c ^H-2d^ donors to C57BL/6 ^H-2b^ recipients or HIF-1α^-/-^ C57BL/6 recipients) and the MHC-matched transplantation models (C57BL/6 ^H-2b^ donors to C57BL/6 ^H-2b^ recipients). The lung allografts and lung isografts were obtained from MHC-mismatched transplantation models and MHC-matched transplantation models, respectively. No immunosuppressive agents or antibiotics were used postoperatively in any mouse model.

### Drug administration

Valproic acid for selective HDAC1 inhibition, RGFP966 for selective HDAC3 inhibition, LMK-235 for selective HDAC4 inhibition, Tubastatin A for selective HDAC6 inhibition, PCI-34051 for selective HDAC8 inhibition, and TSA for pan-HDAC inhibition were purchased from Selleckchem (Houston, TX, USA) and were used in various treatment groups. The chemicals were dissolved in dimethyl sulfoxide (DMSO; Sigma-Aldrich, St. Louis, MO, USA). Vehicle (DMSO) was used in control groups. 2 d before lung allograft transplant, the lung allograft recipient mice were treated with the vehicle, Valproic acid (300 mg/kg) 70,71, RGFP966 (10 mg/kg) 72,73, LMK-235 (20 mg/kg) 74, Tubastatin A (30 mg/kg) 75, PCI-34051 (40 mg/kg) 76, or TSA (1 mg/kg) 77 by intraperitoneal injection every day until lung allograft loss which was determined by Micro-Computer Tomography (micro-CT) Scans, unless specified.

### Imaging, histology, and evaluation of lung graft function

Recipient mice were anesthetized by inhalation of isoflurane (2%), and micro-CT scans (GE Healthcare, Mississauga, ON, Canada) were performed to assess lung graft viability daily. Full consolidation of the lung grafts was defined as the loss of the grafts 8. Synchronously, other lung transplant recipients were sacrificed on postoperative days (POD) 3, 5, and 7. The lower portion of the lung grafts was processed with paraformaldehyde fixation and paraffin embedding. Three sections stained with H&E from the lung grafts were examined, and the grading of the rejection pathology was performed in a blinded fashion using standard criteria for clinical lung transplantation 78. Briefly, using the mononuclear inflammation around the blood vessels and airways, the degree of acute rejection was classified as A0 to A4 (A0=0, none; A1=1, minimal; A2=2, mild; A3=3, moderate; A4=4, severe). For evaluation of the lung graft function, anesthetized recipient mice were administered with mechanical ventilation of 100% FiO_2_ for 3 min. Subsequently, the hilum of the right native lungs was occluded using vascular clamps. After 3 min of ventilation, arterial blood was withdrawn from the left ventricle using a 1 mL heparin-coated syringe for arterial blood gas measurement 79.

### Th17 cell differentiation *in vitro*

Naïve CD4^+^ T cells from C57BL/6 wild-type or C57BL/6 HIF-1α^-/-^mice were purified from a pool of splenocytes using a CD4^+^ T cell isolation kit and an autoMACS cell sorter (Miltenyi Biotec, Bergisch Gladbach, Germany). Naïve CD4^+^ T cells were stimulated with anti-CD3/CD28 beads at a cell: bead ratio of 5: 1. For Th17 cell differentiation, cultures were supplemented with 10 µg/mL anti-IL-12, 10 µg/mL anti-IL-4, 10 µg/mL anti-IFN-γ, 2 ng/mL TGF-β1, 20 ng/mL IL-23, and 20 ng/mL IL-6 (Th17-skewing conditions). The cells were collected on day 5 for further analysis 64, 80. Naïve CD4^+^ T cells in culture were pretreated with Tubastatin A (10 μM) or vehicle (DMSO) for 24 h and then exposed to Th17-skewing conditions to compare the effects of HDAC6i on Th17 cell differentiation.

### Quantitative real-time polymerase chain reaction (qRT-PCR)

Gene expression levels were quantified by qRT-PCR, which was performed with an ABI Prism 7900 Sequence Detection System (Applied Biosystems, Foster City, CA, USA). The data were normalized relative to the expression of glyceraldehyde-3-phosphate dehydrogenase (GAPDH).

### Immunoblot analysis

Western blotting was performed to evaluate the protein expression of HIF-1α. The immunoblot analysis was performed as described previously 81. Briefly, protein lysates from the lung allograft tissues and cell cultures were subjected to sodium dodecyl sulfate-polyacrylamide gel electrophoresis and then transferred to nitrocellulose membranes. The membranes were incubated with anti-HIF-1α (1:1000, Cayman Chemicals, Ann Arbor, Mi, USA), anti-GAPDH, or anti-β-actin (1:1000, Sigma-Aldrich, St. Louis, Mo, USA) overnight at 4 °C. The horseradish peroxidase-labeled secondary antibodies (1:2000) were added and incubated for 1 h at room temperature. Protein bands were detected with the enhanced chemiluminescence western blotting detection system.

### Flow cytometry analysis

Cell cultures and the splenocytes and lung lymphocytes which were isolated from lung graft transplantation models were restimulated with PMA (0.25 mg/mL) and ionomycin (0.25 mg/mL) for 5 h and with brefeldin A (5 mg/mL) for 4 h, and then stained for surface CD4 and CD25. The cells were then fixed, permeabilized, and stained for IL-17A or FoxP3. Flow cytometry data were collected on a FACSCaliber instrument (BD Bioscience, San Jose, CA, USA) and analyzed with the FlowJo software (Tree Star Inc, San Carlos, CA, USA).

### Cytometric bead array (CBA) detection

The serum of recipients was collected, and levels of cytokines IL-17A, IL-6, IL-10, and TGF-β were measured using a Cytometric Bead Array Detection Kit (BD Biosciences, San Diego, CA, USA) according to the manufacturer's protocol.

### Statistical analysis

Continuous variables were expressed as mean ± SD. Comparisons between groups were made with the Student's t-test. Differences among multiple groups were analyzed with one-way ANOVA followed by the Scheffe test. Survival analyses of the grafts were performed using the Kaplan-Meier method. The log-rank test was used to compare differences in survival. The *P*-value of less than 0.05 was considered to indicate a significant difference. The statistics were performed using the SPSS 22.0 Statistics Software Package (IBM Corp., Armonk, NY, USA).

## Results

### HDAC expression is upregulated in acute lung allograft rejection in mice

It is not known whether HDACs are expressed in lung allografts. Our previous studies indicated that lesions of acute rejection were detectable in the lung allografts beginning on POD 3. Although the tissue structures of lung allografts were relatively preserved, acute rejection peaked on POD 7 (8). Therefore, the measurement time points of HDACs expression were selected on POD 3 and POD 7. Except for HDAC3, mRNAs for HDAC1, HDAC4, HDAC6, and HDAC8 were significantly upregulated in lung allografts compared with native lungs of allograft recipients and lung isografts on POD 7 (*P*<0.05). The expression of these HDACs in lung allografts on POD 7 was significantly higher than in lung allografts on POD 3 (*P*<0.05). Notably, higher levels of HDAC6 mRNA were present in the lung allografts on both POD 3 and 7 (*P*<0.05).

### HDAC6-specific inhibitor Tubastatin A attenuates the pathology of acute lung allograft rejection

**Figure 2:**
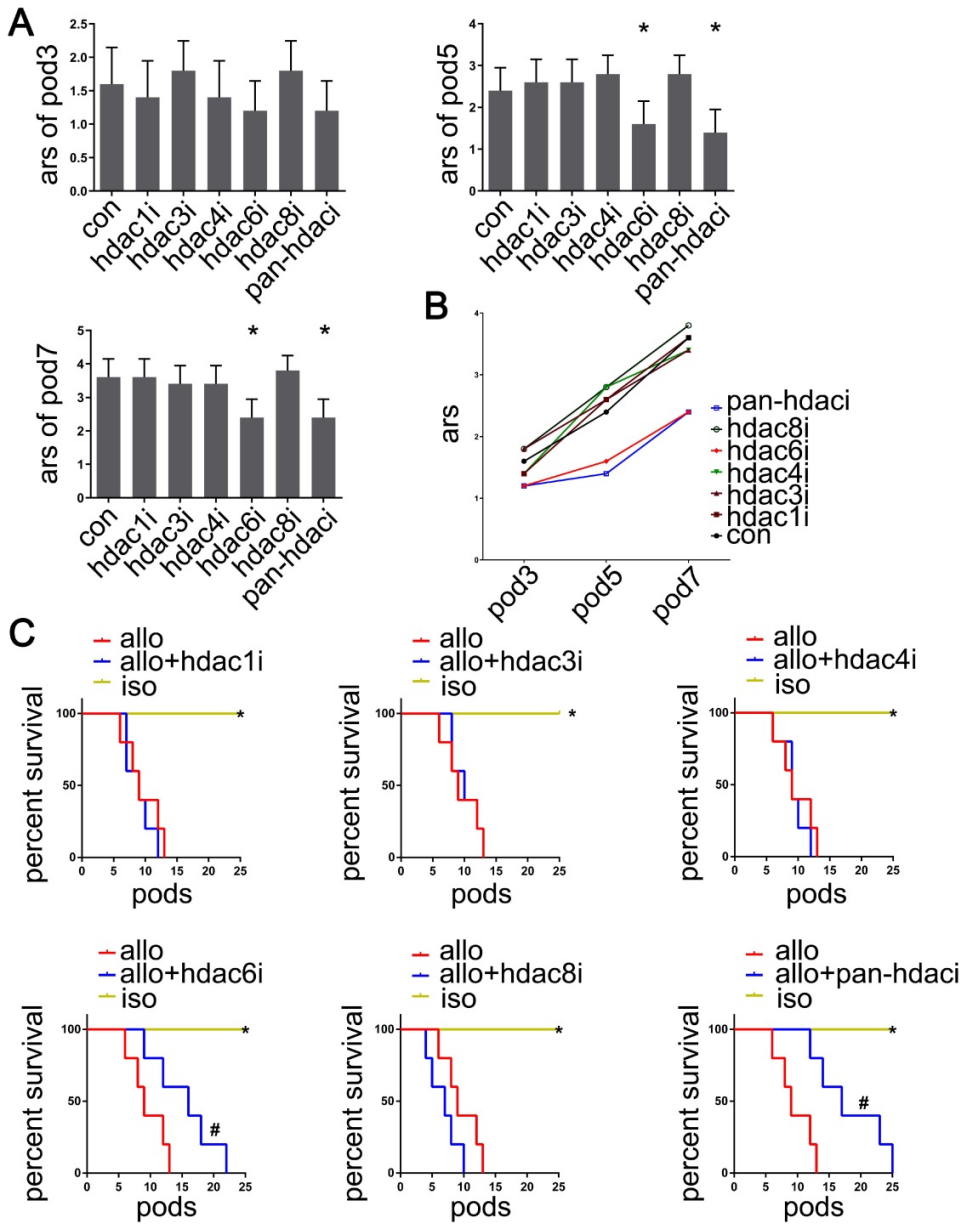
Effects of HDAC inhibitor administration on lung allografts. Based on the pathological and imaging data, acute rejection scores of lung allografts were analyzed by using the standard criteria for clinical lung transplantation for HDAC-specific inhibitors (Valproic acid, RGFP966, LMK-235, Tubastatin A, and PCI-34051 for selective inhibition of HDAC1, HDAC3, HDAC4, HDAC6, and HDAC8, respectively), pan-HDAC inhibitor (TSA), and the vehicle treatment recipients on POD 3, 5, and 7. Data are expressed as mean ± standard deviation, each group at a single time point n=5. *: vs. control, p<0.05 (A and B) The survival analyses were compared among lung allografts of HDAC-specific inhibitor treatment recipients, vehicle treatment recipients, and lung isografts; each group n=15. *: HDAC-specific inhibitor-treated lung allograft recipients vs. lung isograft recipients, p<0.05, vehicle-treated lung allograft recipients vs. lung isograft recipients, p<0.05; #: HDAC-specific inhibitor-treated lung allograft recipients vs. vehicle-treated lung allograft recipients, p<0.05 (C) con: vehicle treatment group; ars: acute rejection scores; pod: postoperative days; hdaci: histone deacetylase inhibitor treatment group; iso: lung isografts in vehicle-treated recipients; allo: lung allografts in vehicle-treated recipients; allo + hdaci: lung allografts in HDACi-treated recipients.

To determine whether pan- or specific HDAC inhibition affected acute lung allograft rejection, we analyzed pathology and imaging data, acute rejection scores, and the survival curves of lung allografts following treatment with the pan-HDAC inhibitor, HDAC-specific inhibitors, and the control vehicle. The results indicated that treatment with the pan-HDAC inhibitor TSA and the HDAC6-specific inhibitor Tubastatin A effectively reduced acute rejection scores of allografts on POD 5 and 7 (*P*<0.05). However, this treatment effect was not observed in HDAC1i-, HDAC3i-, HDAC4i- and HDAC8i-treated recipients (Figure 2A, B). Also, TSA and Tubastatin A caused the lung allografts to survive longer in the treated recipients compared with the vehicle-treated controls (*P*<0.05) (Figure 2C).

**Figure 3:**
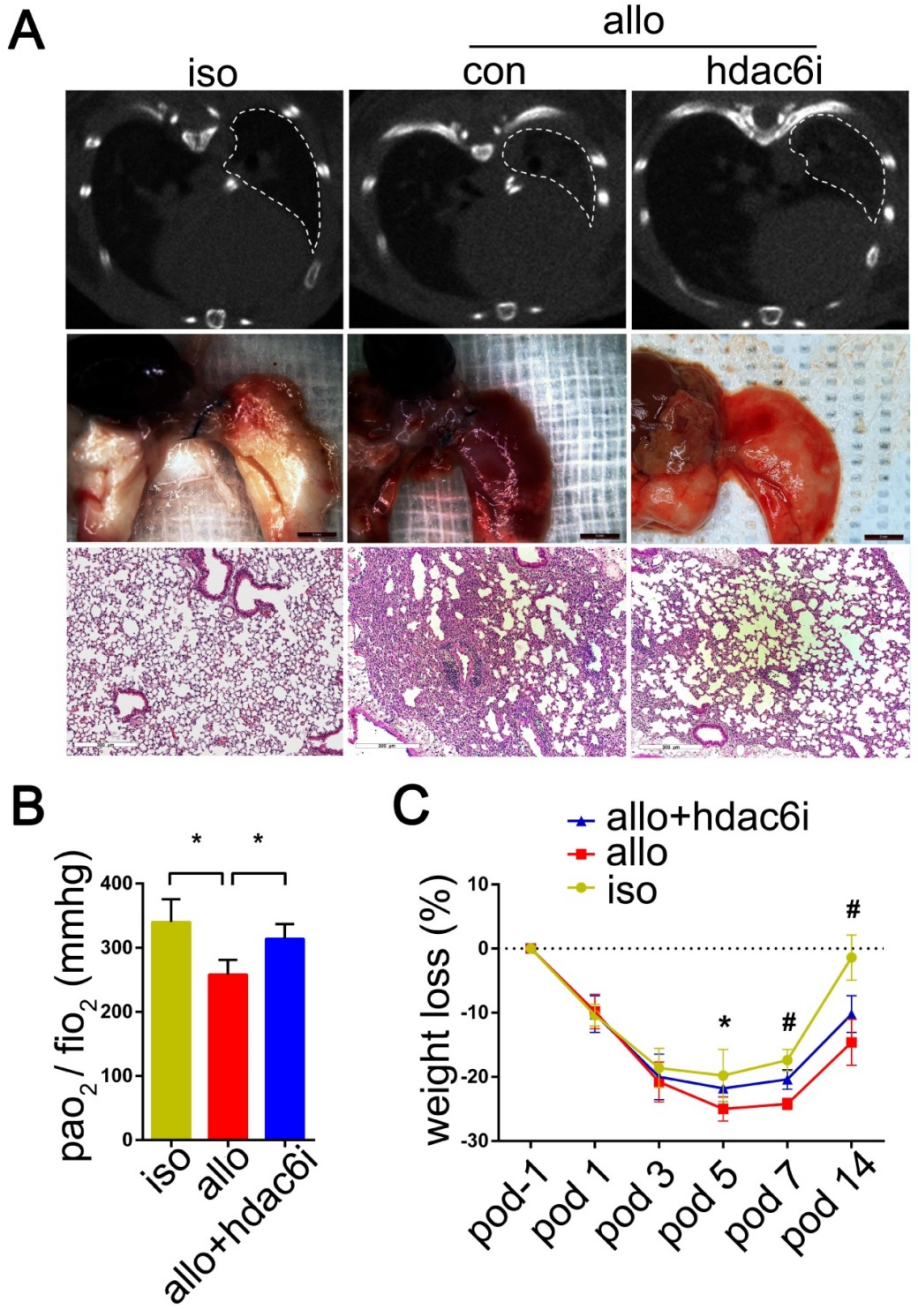
Representative imageology and histopathology of the lung isografts, allografts, and HDAC6-specific inhibitor Tubastatin A-treated allografts; Effects of Tubastatin A administration on lung allograft function (PO_2_/FiO_2_) and weight loss in recipients. Pathological lesions of acute rejection in the lung isografts and allografts of control recipients and allografts of Tubastatin A-treated recipients were evaluated by micro-CT scan, gross pathology, and H&E staining (magnifications: 100×) on POD 5. White dashed line range: lung graft; iso: lung isograft in the vehicle-treated recipient; allo: lung allograft in the vehicle-treated recipient or Tubastatin A-treated recipient (A) The function of lung grafts was assessed by the arterial blood gas measurement from the lung isograft, allograft, and Tubastatin A-treated allograft recipients on POD 5. Data are expressed as mean ± standard deviation, each group n=5. *: p<0.05. po_2_: partial pressure of oxygen; fio_2_: fraction of inspired oxygen (B) Weight loss of recipients was recorded from POD -1 to POD 14. Groups n=5. *: lung isograft recipients vs. lung allograft recipients, p<0.05; #: lung isograft recipients vs. lung allograft recipients, p<0.05, lung allograft recipients vs. Tubastatin A-treated lung allograft recipients, p<0.05, and lung isograft recipients vs. Tubastatin A-treated lung allograft recipients, p<0.05. Data are expressed as mean ± standard deviation (C) iso: vehicle-treated lung isograft recipients; allo: vehicle-treated lung allograft recipients; allo+hdac6i: Tubastatin A-treated lung allograft recipients.

Micro-CT scans showed that lung allografts in the control group displayed a more extensive range of consolidation areas on POD 5 than lung allografts in the Tubastatin A treatment group (Figure 3A). Consistent with this observation, gross pathology revealed a wide range of edema, consolidation, and even bleeding in lung allografts of the control group on POD 5 (Figure 3A), whereas Tubastatin A administration significantly reduced these lesions (Figure 3A). H&E staining of the lung allografts showed different degrees of mononuclear inflammation in the Tubastatin A treatment and control groups. On POD 5, mononuclear inflammation was observed not only around the small airways of control lung allografts but also around the blood vessels and even in the alveolar space. Tubastatin A administration effectively reduced mononuclear inflammation, which was mostly present around the small airways, while aggregation around the blood vessels and in the alveolar space was significantly reduced (Figure 3A) when the arterial blood gas was measured to assess the function of lung grafts under mechanical ventilation. Tubastatin A administration improved the PaO_2_/FiO_2_ of the recipients (*P*<0.05) (Figure 3B). Finally, Tubastatin A-treated lung allograft recipients showed milder weight losses than vehicle-treated lung allograft recipients on POD 7 and 14 (*P*<0.05) (Figure 3C). These results indicated that TSA and Tubastatin A directly attenuate acute allograft rejection.

### Tubastatin A suppresses Th17 cell differentiation *in vitro* and Th17 cell accumulation in the lung transplantation models

To determine whether HDAC6 affects the expression of the Th17 cells in lung transplantation, we first used naïve CD4^+^ T cells to validate HDAC6 activity following 24 h of treatment with 0.1, 1, 5, and 10 μM Tubastatin A. There was a significant effect of the treatment on HDAC6 activity in naïve CD4^+^ T cells for the described conditions. HDAC6 activity decreased in a dose-dependent manner 24 h after Tubastatin A treatment (*P*<0.05) (SI Appendix, Figure S1A). Next, we evaluated the effect of Tubastatin A on naïve CD4^+^ T cell viability, which was not significantly different from high dose Tubastatin A-treated cells (10 μM and 5 μM) compared with low dose Tubastatin A treatment (1 μM) (SI Appendix, Figure S1B). We also found that HDAC6 mRNA expression of naïve CD4^+^ T cells was not affected by treatment with different doses of Tubastatin A (SI Appendix, Figure S1C). These data indicated that Tubastatin A represses HDAC6 activity in naïve CD4^+^ T cells and does not affect cell viability in high concentrations up to 10 μM.

**Figure 4:**
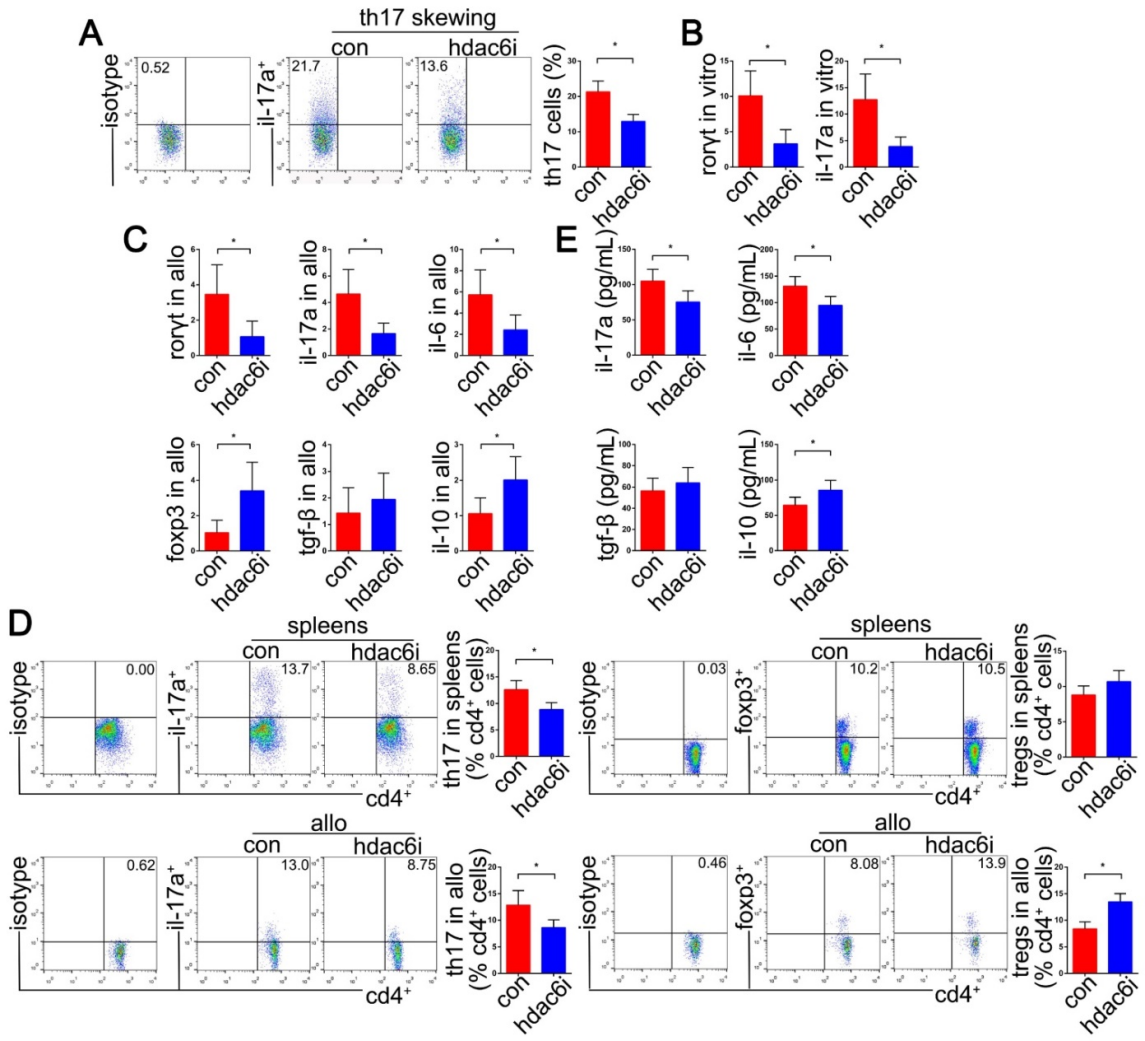
Effects of **HDAC6i Tubastatin A administration on Th17 cell function *in vitro* and *in vivo.*** Naïve CD4^+^ T cells were cultured under Th17-skewing conditions with or without Tubastatin A for 5 d. The dot-plots and bar chart showed the frequencies of Th17 cells in CD4^+^ T cells detected by flow cytometry (A) RORγt and IL-17A mRNAs were detected by qRT-PCR (B) *In vivo*, mRNA levels of RORγt, IL-17A, IL-6, FoxP3, TGF-β, and IL-10 were analyzed by qRT-PCR in the lung allografts of Tubastatin A-treated or vehicle-treated recipients on POD 5 (C) Dot-plots and bar charts show the frequencies of Th17 and regulatory T (Treg) cells in CD4^+^ T cells detected in the spleens and lung allografts of vehicle-treated recipients and Tubastatin A-treated recipients by flow cytometry on POD 5 (D) Sera from Tubastatin A-treated and vehicle-treated recipients were used for testing the pro-inflammatory cytokine levels of IL-17A and IL-6, and anti-inflammatory cytokine levels of TGF-β and IL-10 by cytometric bead array detection on POD 5 (E) For A and B, con: naïve CD4^+^ T cells in Th17-skewing conditions; hdac6i: naïve CD4^+^ T cells in Th17-skewing conditions with Tubastatin A administration. For C-E, con: lung allograft recipients with vehicle treatment; hdac6i: lung allograft recipients with Tubastatin A administration. All bar charts are expressed as mean ± standard deviation. Data represent 3 independent experiments *in vitro* and each group n=5 for experiments *in vivo***.** *: p<0.05. Th17-skewing conditions: cell culture media contained a mixture of anti-IL-12, anti-IL-4, anti-IFN-γ, TGF-β1, IL-23, and IL-6.

Furthermore, we evaluated whether HDAC6i could affect the differentiation efficiency of naïve CD4^+^ T cells into Th17 cells under Th17-skewing conditions *in vitro*. Naïve CD4^+^ T cells were activated in the presence of TGF-β and IL-6, which dramatically promoted Th17 cell differentiation and IL-17A accumulation. HDAC6 activity of naive CD4^+^ T cells was significantly repressed by using 10 μM Tubastatin A. Also, 10 μM Tubastatin A treatment did not significantly affect cell viability compared with 1 μM and 5 μM. These results provided pharmacological and toxicological evidence for pretreatment of naive CD4^+^ T cells with Tubastatin A 24 h before Th17 cell differentiation induction. We found that pretreating naïve CD4^+^ T cells with Tubastatin A (10 μM) significantly suppressed naïve CD4^+^ T cell differentiation into Th17 cell under the Th17-skewing conditions (*P*<0.05), as well as the expression of IL-17A and RORγt mRNA (*P*<0.05) (Figure 4A, B). Moreover, the mRNA expression of FoxP3 was significantly upregulated in Tubastatin A-treated naïve CD4^+^ T cells under Th17-skewing conditions (*P*<0.05) (SI Appendix, Figure S2). However, this phenomenon was not observed in IFN-γ and IL-4 expression (SI Appendix, Figure S2).

The upregulation of Th17 cells in the lung transplant environment has been documented 4,7,8,82,83. We performed the analysis of Th17 cell function by intracellular staining and detection of relevant transcripts and cytokine levels on Tubastatin A-treated models of lung allograft transplantation. Compared with the control group, we observed significant decreases of RORγt, IL-17A, and IL-6 mRNAs in the allografts of the Tubastatin A treatment group on POD5 (*P*<0.05). Strikingly, the pattern of FoxP3, the critical transcription factor of Treg cells, and IL-10 were upregulated (*P*<0.05) (Figure 4C). Consistent with the effect of HDAC6i administration on Th17 cell differentiation *in vitro*, a significant decline of IL-17A^+^ subset of CD4^+^ T cells was detected in the spleens and lung allografts of Tubastatin A-treated recipients (*P*<0.05) (Figure 4D). Furthermore, we also noted a dramatic increase in the proportion of FoxP3^+^ subset of CD4^+^ T cells isolated from the lung allografts of Tubastatin A-treated recipients (*P*<0.05) (Figure 4D).

The serum levels of pro-inflammatory cytokines such as IL-17A and IL-6 were much lower in Tubastatin A-treated recipients than those in the control recipients (*P*<0.05). However, the expression of the anti-inflammatory cytokine, IL-10, was upregulated (*P*<0.05) (Figure 4E). These results suggested that HDAC6i treatment specifically affects the Th17 cell differentiation *in vitro* and Th17 cell accumulation in the lung transplantation models.

### Exogenous IL-17A supplementation eliminates the protective effect of Tubastatin A on lung allografts

**Figure 5:**
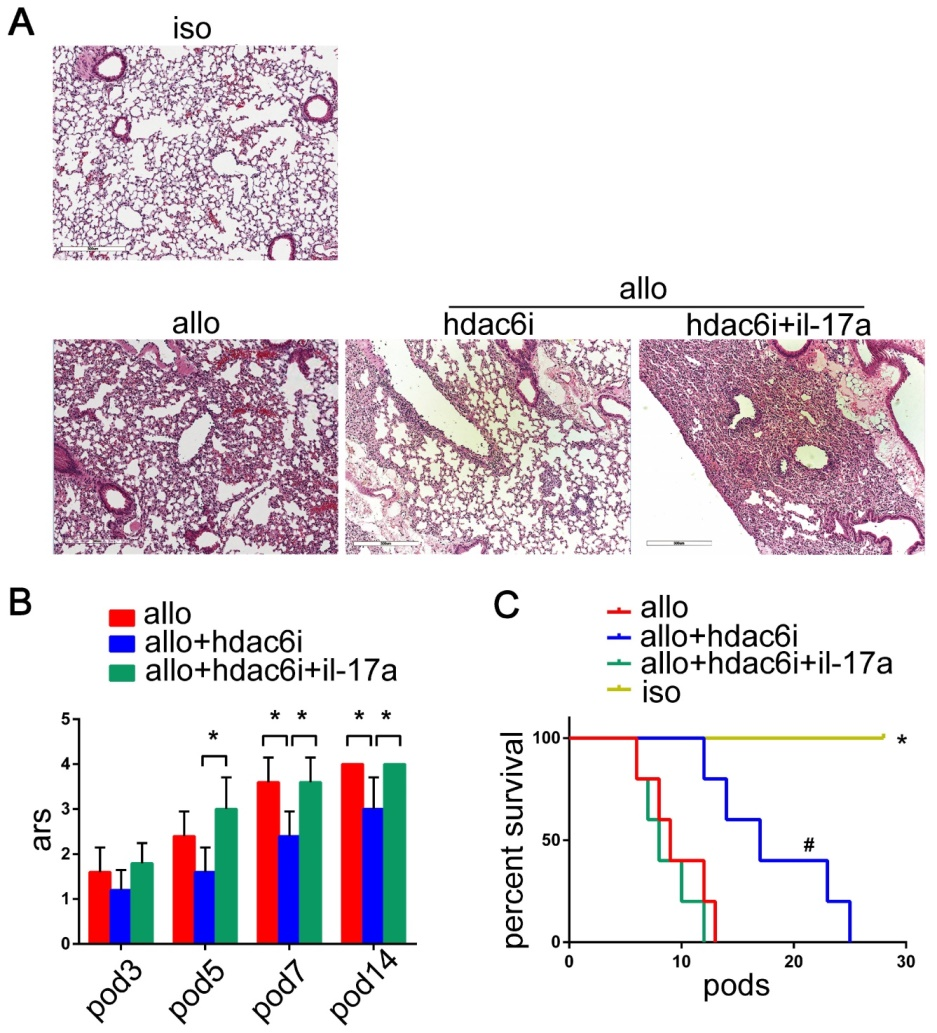
Effects of IL-17A supplementation on acute lung allograft rejection in Tubastatin A-treated recipients. Representative histopathologies of H&E staining (magnifications: 100×) showed pathological lesions of acute rejection in the lung isografts and allografts in **vehicle**-treated recipients, lung allografts in Tubastatin A-treated recipients, and lung allografts in recipients with Tubastatin A treatment plus IL-17A supplementation on POD 5 (A) Acute rejection scores were compared among the lung allografts in the vehicle-treated and Tubastatin A-treated recipients, and those receiving Tubastatin A treatment plus IL-17A supplementation on POD 3, 5, and 7. Data are expressed as mean ± standard deviation, each group at a single time point n=5. *: p<0.05 (B) Survival analyses were compared among the lung isografts, allografts in vehicle-treated and Tubastatin A-treated recipients, and those receiving Tubastatin A treatment plus IL-17A supplementation. Each group n=15. *: lung allografts in Tubastatin A-treated recipients vs. lung isografts, p<0.05, lung allografts in recipients with Tubastatin A treatment plus IL-17A supplementation vs. lung isografts**,** p<0.05, and lung allografts in vehicle-treated recipients vs. lung isografts, p<0.05; #: lung allografts in Tubastatin A-treated recipients vs. lung allografts in recipients with Tubastatin A treatment plus IL-17A supplementation, p<0.05, lung allografts in Tubastatin A-treated recipients vs. lung allografts in vehicle-treated recipients, p<0.05 (C) iso: lung isografts; allo: lung allografts in vehicle-treated recipients; allo+hdac6i: lung allografts in Tubastatin A-treated recipients; allo+hdac6i+il-17a: lung allografts in recipients with Tubastatin A treatment plus IL-17A supplementation.

Although we established the role of HDAC6 in the differentiation of Th17 cells *in vitro* and the expression of Th17 cells in the lung transplantation models, it was unclear whether HDAC6i protected lung allografts by downregulating the function of Th17 cells. We supplemented IL-17A in lung allograft recipients after Tubastatin A treatment to investigate the role of Th17 cell function regulation in Tubastatin A-mediated attenuation of acute lung allograft rejection. First, we administered recombinant mouse IL-17A (300 ng/mouse, i.v) 84 (PeproTech, Rocky Hill, NJ, USA) to C57 mice, and detected the concentration of IL-17A in the peripheral blood by CBA at 6 and 24 h after IL-17A injection. The results showed that, compared to the control group, peripheral blood IL-17A concentration in the exogenous IL-17A treatment group significantly increased (SI Appendix, Figure S3). However, 24 h after injection, IL-17A concentration in the peripheral blood of exogenous IL-17A-treated mice was equivalent to 1/3 of that in the peripheral blood of lung allograft recipients (SI Appendix, Figure S3). Based on these results, exogenous IL-17A of 300 ng/mouse was defined as the “low dose”, which was supplemented on POD 2 and 4 with Tubastatin A treatment in the lung allograft recipients. Pathological analysis showed that the lung allografts of Tubastatin A treatment plus IL-17A-supplemented group exhibited more severe mononuclear inflammation than observed in the lung allografts of Tubastatin A treatment alone group (Figure 5A). Blinded pathologic scoring revealed significantly higher grades of acute rejection for the lung allografts in IL-17A-supplemented recipients (*P*<0.05) (Figure 5B). Furthermore, a low dose of IL-17A shortened lung allograft survival in the Tubastatin A plus IL-17A-treated recipients compared with Tubastatin A treatment only recipients (*P*<0.05) (Figure 5C). These findings indicated that IL-17A eliminates the anti-rejection effect of HDAC6i.

### HIF-1α is overexpressed in lung allografts

**Figure 6:**
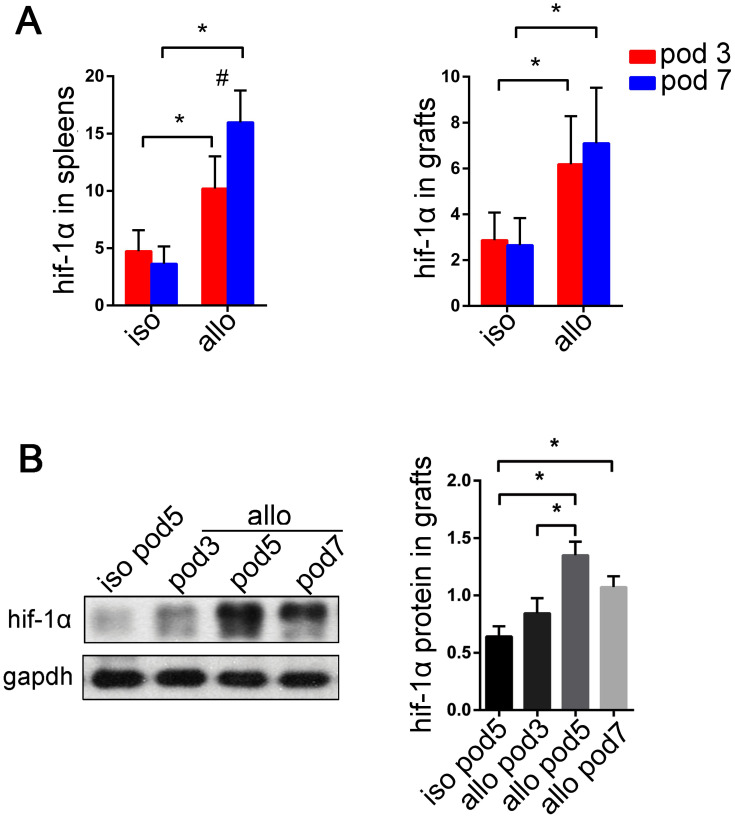
Expression of HIF-1α transcripts in lung grafts and spleens, and protein levels in lung grafts. The spleens and lung grafts from isograft and allograft recipients were analyzed for mRNA expression of HIF-1α by qRT-PCR on POD 3 and 7. Data are expressed as mean ± standard deviation, each group at a single time point n=5. *: p<0.05; #: HIF-1α mRNA in spleens of allograft recipients on POD 3 vs. HIF-1α mRNA in spleens of allograft recipients on POD 7, p<0.05 (A). Representative western blot image and the bar chart showed the protein levels of HIF-1α in lung isografts on POD 5 and in lung allografts on POD 3, 5, and 7. The protein levels of HIF-1α were normalized to the GAPDH levels. Data are expressed as mean ± standard deviation, each time point n=3. *: p<0.05 (B). hif-1α: hypoxia-inducible factor; iso: lung isograft recipients; allo: lung allograft recipients; gapdh: glyceraldehyde-3-phosphate dehydrogenase.

The HIF-1α expression was reported to be induced in the T cells during Th17 cell differentiation in both hypoxia and normoxia 62-69. We found that the expression of HIF-1α and HDAC6 mRNAs was significantly upregulated in mouse naïve CD4^+^ T cells, human bronchial epithelial cells (BEAS-2B), and human pulmonary microvascular endothelial cells (HPMECs) *in vitro* under Th17-skewing conditions for 5 d. (SI Appendix, Figure S4). However, little is known about the appearance of HIF-1α in the lung allografts and recipients. In our study, we observed HIF-1α mRNA in both isograft and allograft groups. The levels of HIF-1α transcripts significantly increased in lung allografts and spleens of the allograft group compared with those of the isograft group (*P*<0.05) (Figure 6A). Also, the lung allografts accumulated HIF-1α protein over time in recipients under the acute rejection background (*P*<0.05) (Figure 6B). These results support the notion that HIF-1α expression is not only increased in Th17 cell differentiation *in vitro*, but is also abnormal in acute lung allograft rejection.

### HIF-1α deficiency suppresses Th17 cell function and attenuates lung allograft rejection

**Figure 7:**
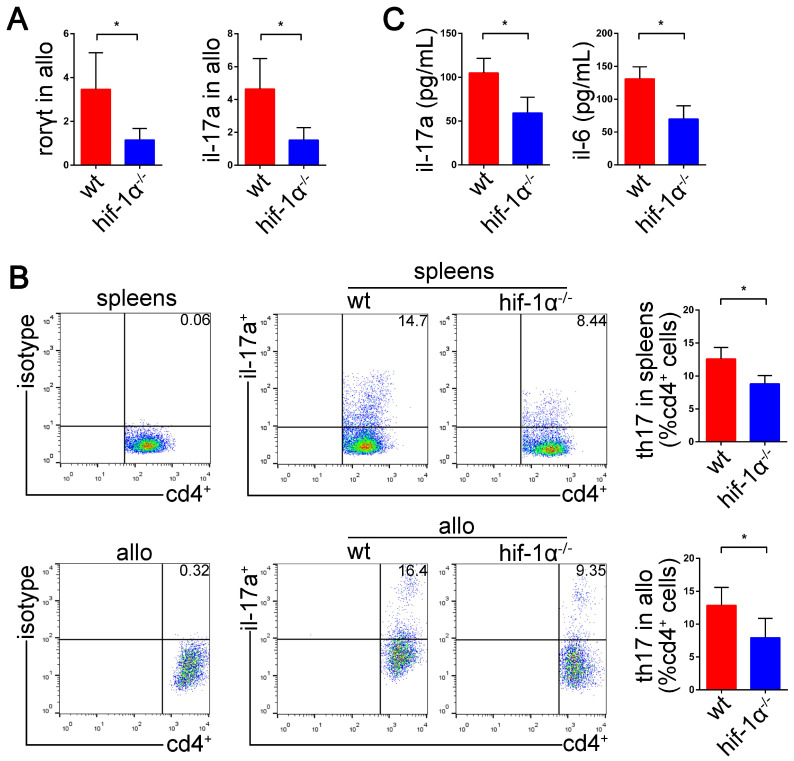
Effects of HIF-1α **deficiency on the function of Th17 cells in the lung allografts.** mRNA expression of RORγt and IL-17A were tested in lung allografts of the wild-type and HIF-1α^-/-^ recipients by qRT-PCR on POD 5 (A) Representative dot-plots and the bar charts showed the frequencies of Th17 cells in CD4^+^ T cells detected in spleens and lung allografts of wild-type and HIF-1α^-/-^ recipients by flow cytometry on POD 5 (B) IL-17A and IL-6 levels in sera of wild-type and HIF-1α^-/-^ recipients tested by using the cytometric bead array method (C) allo: lung allografts; wt: wild-type recipients; hif-1α^-/-^: HIF-1α^-/-^ recipients. All bar charts are expressed as mean ± standard deviation, each group n=5. *: p<0.05.

We have demonstrated high expression of HIF1-1α and RORγt in acute lung allograft rejection. Next, we explored whether HIF-1α deficiency downregulated RORγt expression and Th17 cell function in lung allograft transplantation. HIF-1α^-/-^ C57BL/6 mice were used as the recipients, and the Th17 cell function was examined by measuring RORγt and IL-17A mRNAs, Th17 cell proportion, and Th17 cell-related inflammatory cytokine levels. As compared with the lung allografts in wild-type recipients, the lung allografts in HIF-1α^-/-^ recipients exhibited decreased RORγt and IL-17A mRNAs (*P*<0.05) (Figure 7A). Also, a reduced percentage of Th17 cells in CD4^+^ T cells was observed in the spleens and lung allografts of HIF-1α^-/-^ recipients (*P*<0.05) (Figure 7B). Furthermore, CBA detection revealed decreased expression of IL-17A and IL-6 in the serum of HIF-1α^-/-^ recipients (*P*<0.05) (Figure 7C). Thus, HIF-1α deficiency resulted in the suppression of RORγt expression and Th17 cell function in lung allograft transplantation.

**Figure 8:**
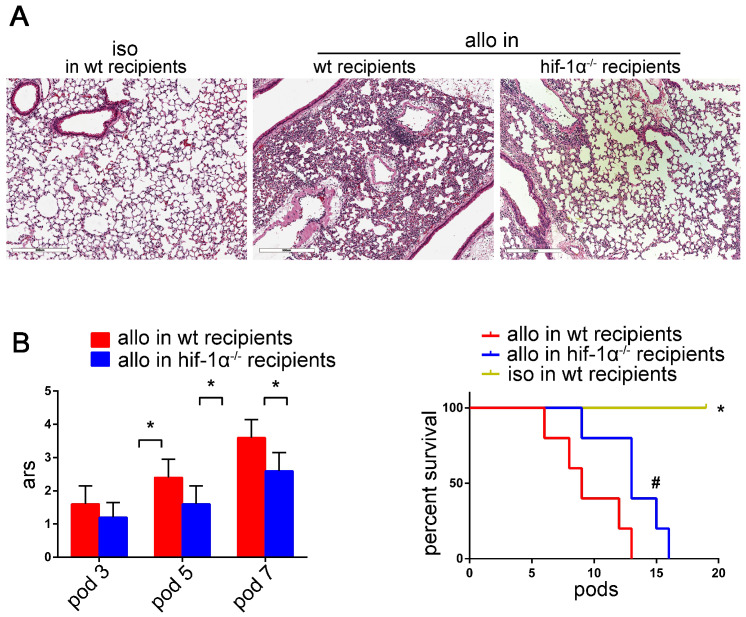
Effects of HIF-1α **deficiency on** acute lung allograft rejection. Representative histopathologies by H&E staining (magnifications: 100×) showed pathological lesions of acute rejection in the lung isografts and allografts in wild-type and HIF-1α^-/-^ recipients on POD 5 (A) Acute rejection scores of lung allografts in wild-type recipients compared with those of HIF-1α^-/-^ recipients on POD 3, 5, and 7. Data are expressed as mean ± standard deviation, each group at a single time point n=5. *: p<0.05 (B) Survival analyses were compared among the lung isografts and allografts in wild-type recipients and the lung allografts in HIF-1α^-/-^ recipients. Each group n=15. *: lung allografts in HIF-1α^-/-^ recipients vs. lung isografts**,** p<0.05, lung allografts in wild-type recipients vs. lung isografts, p<0.05; #: lung allografts in HIF-1α^-/-^ recipients vs. lung allografts in wild-type recipients, p<0.05 (C) iso: lung isografts; allo: lung allografts; wt: wild-type.

As shown in figure 8A, lung allografts of HIF-1α^-/-^ recipients exhibited remarkable preservation of lung architecture compared with the lung allografts of wild-type recipients. Besides, significant decreases of acute rejection scores were observed in the lung allografts of HIF-1α^-/-^ recipients compared with those of the wild-type recipients (*P*<0.05) (Figure 8B). HIF-1α deficiency of the recipients permitted the lung allografts to survive longer (*P*<0.05) (Figure 8C). Taken together, these data suggested that HIF-1α plays a critical role in Th17 cell function and contributes to Th17-dependent acute lung allograft rejection.

### Tubastatin A downregulates HIF-1α transcriptional activity in Th17-skewing conditions and promotes HIF-1α protein degradation in lung allografts

**Figure 9:**
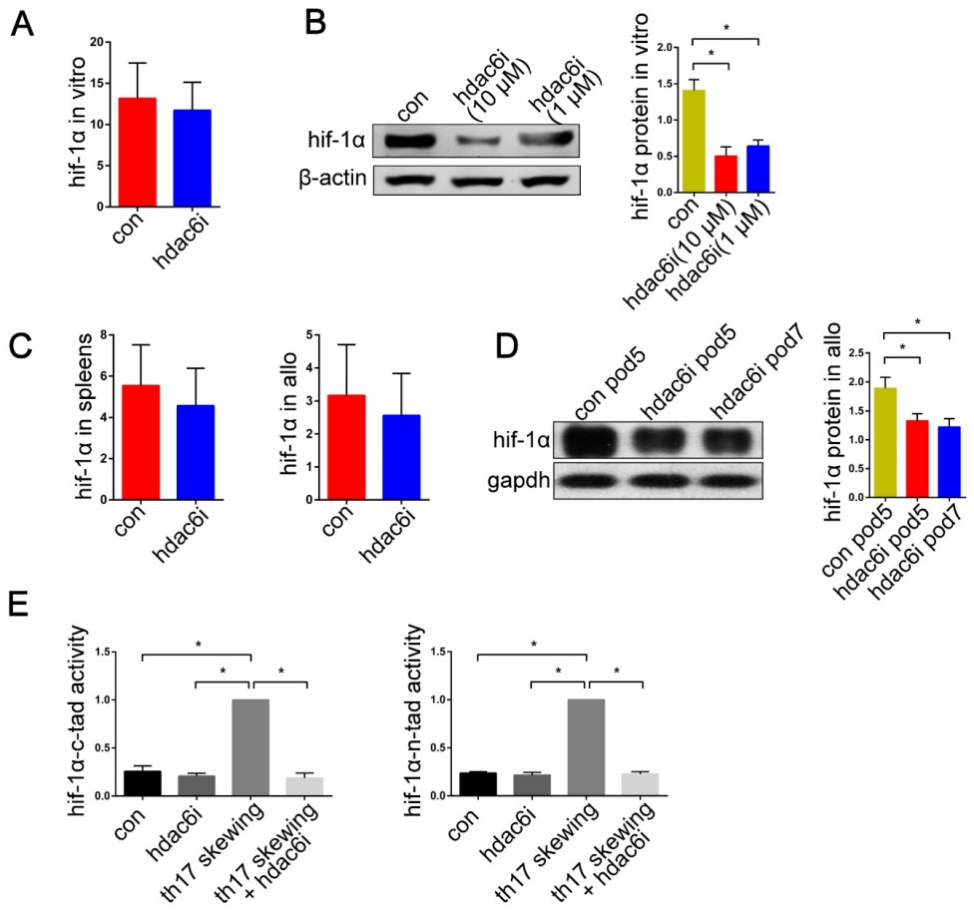
Effects of **HDAC6i Tubastatin A administration on HIF-1**α function *in vitro* and *in vivo.* Naïve CD4^+^ T cells were cultured under Th17-skewing conditions with or without Tubastatin A treatment for 5 d and HIF-1α mRNA expression was measured (A) Representative western blot image and the bar charts show protein levels of HIF-1α in naïve CD4^+^ T cells cultured under Th17-skewing conditions with or without Tubastatin A treatment for 5 d. HIF-1α protein expression was normalized to the β-actin levels. Data represent 3 independent *in vitro* experiments (B) The spleens and lung allografts in vehicle-treated and Tubastatin A-treated recipients were collected for the measurement of HIF-1α mRNA levels on POD 5. Each group n=5 (C) Representative western blot image and the bar charts show HIF-1α protein levels in lung allografts of **vehicle-treated** recipients on POD 5 and the lung allografts of Tubastatin A-treated recipients on POD 5 and 7. HIF-1α protein expression was normalized to the GAPDH levels. Each time point n=3 (D). HIF-1α-N-TAD and HIF-1α-C-TAD luciferase activities were analyzed for measuring HIF-1α activity in the naïve CD4^+^ T cells with or without Tubastatin A treatment and the naïve CD4^+^ T cells under Th17-skewing conditions with or without Tubastatin A. The HIF-1α luciferase activity is presented as the fold change relative to HIF-1α luciferase activity in naïve T cells under Th-17 skewing conditions. Data represent 3 independent experiments *in vitro* (E) For A and B, con: naïve CD4^+^ T cells were cultured under Th17-skewing conditions for 5 d; hdac6i: naïve CD4^+^ T cells were cultured under Th17-skewing conditions with Tubastatin A for 5 d. For C and D, con: lung allograft recipients of vehicle treatment; hdac6i: lung allograft recipients of Tubastatin A treatment. For E, con: naïve CD4^+^ T cells; hdac6i: Tubastatin A-treated naïve CD4^+^ T cells; th17 skewing: naïve CD4^+^ T cells under Th17-skewing conditions; th17 skewing + hdac6i: naïve CD4^+^ T cells under Th17-skewing conditions with Tubastatin A administration; tad: transactivating domain. All bar charts are expressed as mean ± standard deviation. *: p<0.05.

It has been shown that selective inhibition of HDAC6 results in decreased HIF-1α activity under both normoxia and hypoxia 59. We, therefore, tested the hypothesis that Tubastatin A regulates the function of HIF-1α and thereby affects the prevalence of Th17 cells in lung allograft recipients. Previous studies showed that the expression of HIF-1α mRNA increased significantly under Th17-skewing conditions 63,64. In the current study, treatment with Tubastatin A had no inhibitory effect on HIF-1α mRNA expression of T cells under Th17-skewing conditions (Figure 9A). We then examined the expression of HIF-1α mRNA in spleens and lung allografts in lung transplantation models. As expected, there was no significant difference between the control and the Tubastatin A group (Figure 9C). However, the protein expression of HIF-1α in naïve CD4^+^ T cells was tested by western blotting. The results showed that HIF-1α protein level was significantly upregulated in naïve CD4^+^ T cells with Th17-skewing treatment compared with naïve CD4+ T cells in normal culture conditions (*P*<0.05) (Figure 9B). Moreover, compared with the control group, Tubastatin A treatment induced downregulation of HIF-1α protein levels in lung allografts (*P*<0.05) (Figure 9D).

Next, we utilized TAD reporters to measure the effect of Tubastatin A on HIF-1α activity *in vitro*. Inhibition of HDAC6 resulted in decreased HIF-1α-N-TAD and HIF-1α-C-TAD activities in the T cells under Th17-skewing conditions (*P*<0.05) (Figure 9E). It is noteworthy that, despite the absence of Tubastatin A, the HIF-1α activity of naïve T cells was much lower than that of naïve T cells under Th17-shewing conditions (*P*<0.05) (Figure 9E). Collectively, these data indicated that under Th17-skewing conditions, HDAC6i downregulates HIF-1α function by affecting HIF-1α activity and HIF-1α protein levels in lung allografts.

### HIF-1α is required for the protective effect of Tubastatin A on acute lung allograft rejection

**Figure 10:**
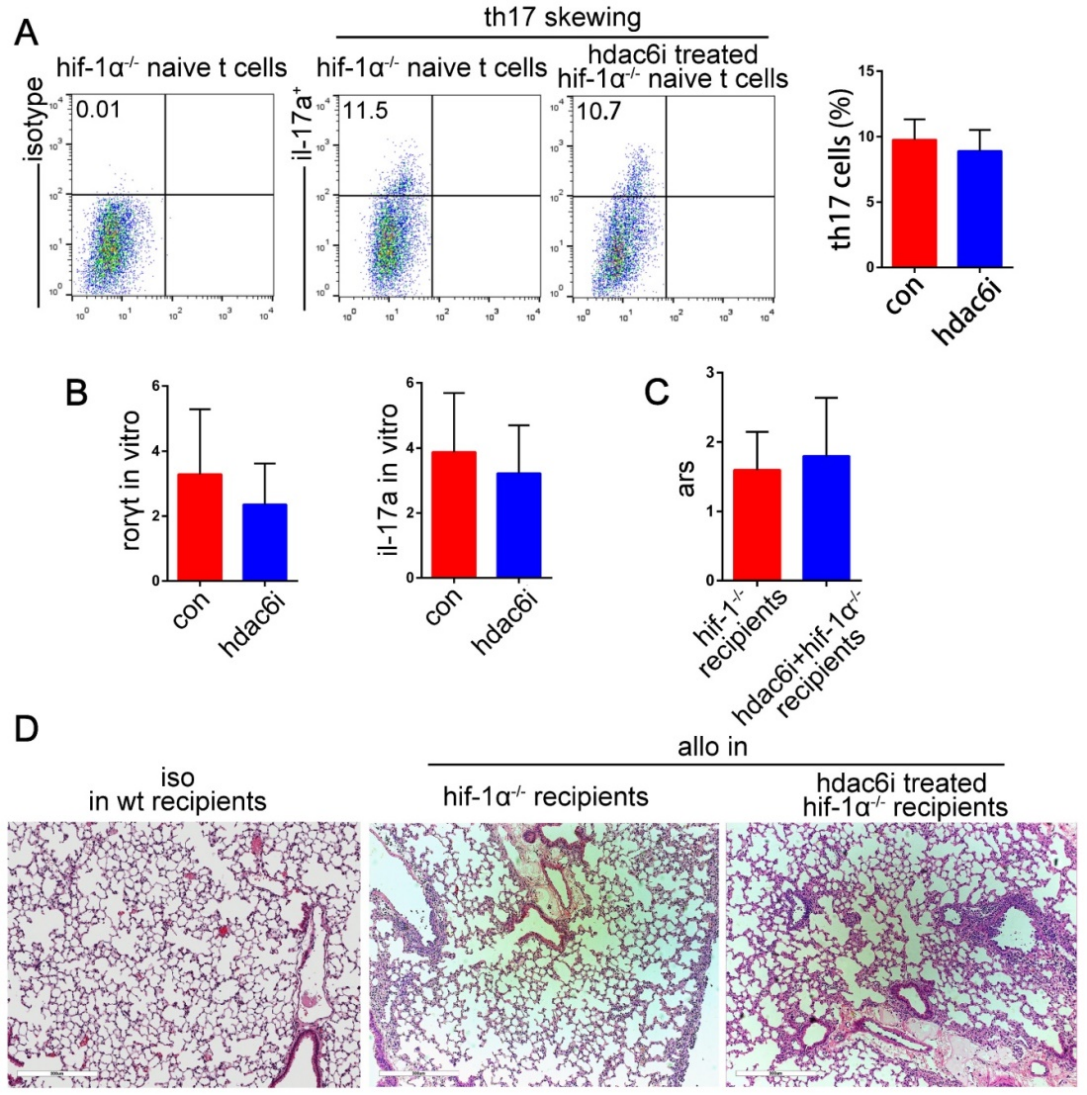
Effects of **HDAC6i** Tubastatin A administration on HIF-1α-/- naïve CD4+ T cell differentiation into Th17 cell and on acute lung allograft rejection in HIF-1α-/- recipients. HIF-1α-/- naïve CD4+ T cells were cultured under Th17-skewing conditions with or without Tubastatin A for 5 d. Dot-plots and bar charts show the frequencies of Th17 cells by flow cytometry (A) mRNA expression of RORγt and IL-17A was analyzed in HIF-1α-/- naïve CD4+ T cells that were cultured under Th17-skewing conditions with or without Tubastatin A (B) Acute rejection scores were assessed in the lung allografts of HIF-1α-/- recipients with or without Tubastatin A treatment on POD 5 (C) Representative histopathologies by H&E staining (magnifications: 100×) show pathological lesions of acute rejection in lung isografts and allografts of HIF-1α-/- recipients with or without Tubastatin A treatment on POD 5 (D) con: HIF-1α-/- naïve CD4+ T cells were cultured under Th17-skewing conditions for 5 d; hdac6i: HIF-1α-/- naïve CD4+ T cells were cultured under Th17-skewing conditions with Tubastatin A for 5 d; iso: lung isografts; allo: lung allografts. Data represent 3 independent experiments *in vitro*. Each group n=5 for experiments *in vivo*.

Our results indicated that both HDAC6i and HIF-1α played critical roles in suppressing Th17 cell function and the attenuation of acute lung allograft rejection. We hypothesized that HDAC6i, which downregulated HIF-1α activity and protein levels, exerted its protective effects on lung allograft rejection via the HIF-1α/ RORγt /Th17 pathway. For further investigation, naïve CD4^+^ T cells were isolated from HIF-1α^-/-^ mice and treated with or without Tubastatin A under Th17-skewing conditions. Flow cytometry analysis indicated that Tubastatin A treatment did not affect the differentiation of HIF-1α^-/-^ naïve CD4^+^ T cells into Th17 cells under Th17-skewing conditions (Figure 10A). Additionally, RORγt and IL-17A mRNA levels were comparable in Tubastatin A-treated and untreated HIF-1α^-/-^ naïve CD4^+^ T cells under inflammatory stimulation (Figure 10B). Consistent with these findings, the pathologic lesions of lung allografts were not attenuated in the HIF-1α^-/-^ recipients of Tubastatin A treatment (Figure 10C,D). Collectively, these data indicated that HDAC6i did not suppress the HIF-1α^-/-^ naïve CD4^+^ T cell differentiation into Th17 cells under Th17-skewing conditions and did not have a protective effect on lung allografts in HIF-1α^-/-^ recipients. Thus, we believe that HIF-1α is essential for HDAC6i-induced Th17 cell downregulation in lung allograft rejection.

## Discussion

Small molecule inhibitors have demonstrated their potential in attenuation of acute lung allograft rejection by regulating T cell function 85,86. Stabilizing Treg cell function may be one of the essential mechanisms for HDACi to play a protective role in organ transplantation 24,25,27,50,77,87,88. We found that HDAC6i upregulated the expression of anti-inflammatory cytokine IL-10 in the peripheral blood of recipients and Treg cells in lung allografts. However, the plasticity of Treg cells was observed in autoimmune and other inflammatory conditions, such as type 1 diabetes, juvenile arthritis, multiple sclerosis, and organ transplantations 38,89-96. Decreased stability of Foxp3 expression and increased numbers of Th1-like IFN-γ^+^ Treg cells and Th17-like IL-17^+^ Treg cells were also detected in these conditions 11,97-103. We, therefore, focused on the direct downregulation of Th17 cell function rather than via promoting Treg cell function.

In this study, we demonstrated that the differentiation and function of Th17 cells were downregulated by the HDAC6-specific inhibitor Tubastatin A* in vitro* and during acute lung allograft rejection. The strategy of using HDAC6i to suppress Th17 cells exhibited protective roles in lung allografts. Moreover, we measured Th17 and Treg cell fractions in the lung allografts and immune organs such as the spleen. The results showed that the expression of Th17 cells was downregulated and Treg cells were upregulated in both lung allografts and spleens. Also, the detection of cytokines in the peripheral blood of lung allograft recipients revealed that IL-17A was significantly downregulated, while IL-10 was significantly upregulated in the Tubastatin A treatment group. These results suggested that Tubastatin A treatment had systemic effects on the functions of Th17 and Treg cells in lung allograft recipients and were not localized to Th17 and Treg cells in the lung allografts. Other types of HDACs, such as HDAC1, HDAC3, HDAC4, and HDAC8, also had high expression in lung allografts, but their specific inhibitors could not effectively alleviate acute lung allograft rejection. The characteristics of the up-regulated expression in HDAC1, HDAC4, HDAC6, and HDAC8 are not the same, which may be related to the different responses of HDACs to inflammatory stress caused by acute lung allograft rejection 104,105.

As regards mechanisms, several pathways could be involved. It has been shown that Th17 polarization was promoted by short-chain fatty acid-induced HDAC inhibition through a p70 S6 kinase-rS6 in T cells 37. Others have identified the IL-6/STAT3/IL-17 pathway as an important target of HDAC inhibitors in experimental colitis 45. In rheumatoid arthritis models, pan-HDACi SAHA was shown to inhibit Th17 cell differentiation through nuclear receptor subfamily 1 group D member 1 42. In the current study, we observed high HIF-1α expression in lung allograft transplantation models. HIF-1α-deficient mice were resistant to acute lung allograft rejection and showed significantly less accumulation of Th17 cells. These results are consistent with earlier observations that HIF-1α expression in T cells can be induced by hypoxic and normoxic stimuli and HIF-1α is required for Th17 cell development 63,64.

Herein, we elucidated a novel pathway by which HDAC6i affected acute lung allograft rejection. We found that Tubastatin A reduced HIF-1α protein level and function. However, Tubastatin A did not affect HIF-1α transcription even though other studies reported that HDAC6i induced HIF-1α transcriptional activity of nucleus pulposus cells *in vitro* independent of HIF-1α protein stability 59. Our research and other studies 106-109 have demonstrated that inflammatory factors promote HIF-1α protein expression. Conceivably, downregulation of HIF-1α protein may be related to the reduction of inflammation levels in Tubastatin A-treated lung transplantation models 110-113. However, we could not exclude other possibilities 114,115, such as the VHL-independent HSP70/HSP90 pathway or the PHD2-dependent mechanism, which could be involved in HDACi-mediated HIF-1α degradation. Finally, we found that, unlike the wild-type mice, Th17 cell function and lung allograft rejection were not downregulated by Tubastatin A in HIF-1α-deficient mice.

Previous studies have identified several mechanisms by which HIF-1α regulated Th17 cell function, such as HIF-1α-dependent glycolytic pathway 64, HIF-1α-miR-210-STAT6/LYN pathway 116, HIF-1α-IL-12p40-controlled Th1/Th17 response 68, and HIF-1α/synovial fibroblasts-mediated expansion of Th17 cells 117. However, other studies have shown that RORγt was an important HIF-1α target that regulated Th17 cell function 62,63,66,118. In our study, we observed that HIF-1α deficiency reduced RORγt expression in the spleen and lung allografts under acute rejection. We believe that the downregulation of RORγt expression is an important mechanism for HIF-1α, causing Th17 cell inhibition when lung allograft rejection occurs.

In summary, our findings indicate that the HDAC6/HIF-1α/ROR γt/Th17-signaling axis plays a critical role in the pathological lesions of acute lung allograft rejection and suggest that HDAC6i, Tubastatin A, has therapeutic effects by targeting this signaling axis.

## Supplementary Material

Supplementary figures and tables.

## Figures and Tables

**Figure 1:**
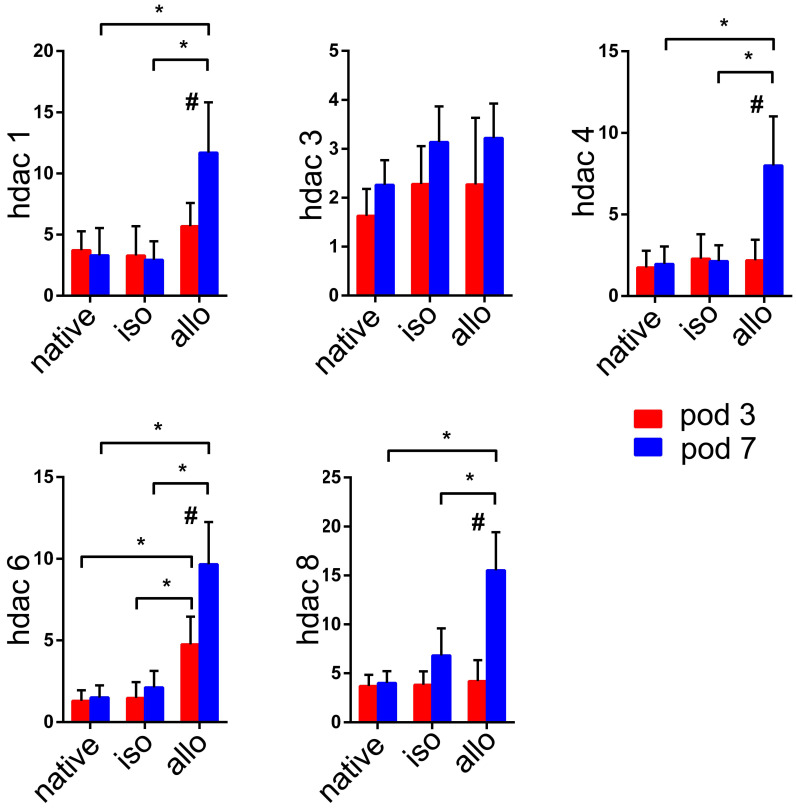
m**RNA expression of histone deacetylases (HDACs) in the native lungs and lung isografts and allografts on postoperative days (POD) 3 and POD 7.** mRNA expression levels of HDACs in the native lungs of allograft recipients, lung isografts, and lung allografts were measured by quantitative real-time PCR (qRT-PCR). The results were normalized to the glyceraldehyde-3-phosphate dehydrogenase (GAPDH) levels. Data are expressed as mean ± standard deviation, each group at a single time point n=5. *: p<0.05; #: lung allografts on POD 3 vs lung allografts on POD 7, p<0.05. hdac: histone deacetylase; native: native lungs; iso: lung isografts; allo: lung allografts; pod: postoperative days.

## Abbreviations

CBA: cytometric bead array
CLAD: chronic lung allograft dysfunction
DMSO: dimethyl sulfoxide
GAPDH: glyceraldehyde-3-phosphate dehydrogenase
HDAC: histone deacetylase
HDAC6i: HDAC6-specific inhibitor
HIF-1α: hypoxia-inducible factor-1α
MHC: major histocompatibility antigen
POD: postoperative days
ROR γt: retinoic acid-related orphan receptor γt
TSA: Trichostatin A
