# Supplementary material for: HDAC6-specific inhibitor suppresses Th17 cell function via the HIF-1α pathway in acute lung allograft rejection in mice
Source: Theranostics. 2020 May 21;10(15):6790–805. doi: 10.7150/thno.44961 (PMC7295069; doi:10.7150/thno.44961)
Supplement: Supplementary file 1 — Supplementary figures and tables. [file thnov10p6790s1.pdf]

Supplemental data *"HDAC6-specific inhibitor suppresses Th17 cell function via the HIF-1α pathway in acute lung allograft rejection in Mice" by Wenyong Z. et al.*

Supplementary Methods

Orthotopic mouse lung transplantation procedure

The donor mouse was anesthetized by inhalation of isoflurane (5%). Subsequently, a 14 mm 20-gauge angiocatheter was inserted into the trachea and connected to the ventilator (settings: O_2: 1 L/min; respiratory rate: 120 breaths/min; tidal volume: 0.5 mL), and the mouse was maintained under inhaled anesthesia (2%). After thoracotomy, the right ventricle was slowly injected with a 3 mL low potassium dextran solution (Perfadex, Medisan, Uppsala, Sweden). The heart-lung block was removed from the mouse and placed in 4 ℃ Perfadex preservation solution. The pulmonary artery (PA) and pulmonary vein (PV) were dissected from the hilum and, together with the bronchus of donor's lung, were cuffed; the cuffs were prepared from 24 to 22-gauge angiocatheter. All surgical procedures of lung grafts were performed on the cold, moist sterile gauze. The cold ischemic time of lung grafts was 35.3 ± 5.4 min (n=30). The recipient mouse was anesthetized in the same way as the donor mouse. The recipient mouse was placed in the right lateral decubitus position and performed the left thoracotomy. The PV and PA of the left lung in recipient mice were dissected from bronchus. After using an aneurysm clip to block the left hilum of the recipient, 9-0 nylon sutures were placed around the PA, PV, and bronchus for future securing of the cuffs. The cuffed donor was transplanted into the recipient through the incisions made on the recipient PA, PV, and bronchus. The donor PA, PV, and bronchus were then inserted into corresponding structures of the recipients. After securing with 9-0 nylon suture, the clip was removed. The lung graft was re-perfused and placed back in the chest. The warm ischemia time of lung grafts was 15.6 ± 4.3 min (n=30). The whole procedure of orthotopic mouse lung transplantation lasted 68.5 ± 7.2 min (n=30). The 24 h survival rate of lung allograft recipients was 90% (n=30). The recipient mouse awoke naturally after cessation of isoflurane inhalation. No immunosuppressive agents or antibiotics were used postoperatively in the recipient mouse.

Evaluation of lung graft function

In this study, we relied on arterial blood gas (ABG) measurements to assess lung graft function [1]. The recipient mice were anesthetized by inhalation of isoflurane (5%), and then a 14 mm 20-gauge angiocatheter was inserted into the trachea and connected to the ventilator (settings: O_2: 1 L/min;

isoflurane: 2%; respiratory rate: 120 breaths/min; tidal volume: 0.5 mL). After thoracotomy, lung graft recipients were mechanically ventilated for both lungs with 100% FiO2 for 3 min. Next, the hilum of the native non-transplanted lung was occluded for 3 min. Finally, the arterial blood of lung graft recipients was drawn from the left ventricle to measure ABG by using a 1 mL heparin-coated syringe with 25 G needle.

Isolation of $CD4^+$ T cells from lung grafts

The lung graft was cut into 1-2 mm^2 pieces. The pieces were transferred into the 15 mL tube containing 10 mL of collagenase digestion solution at 37 ℃ for 1 h under constant horizontal shaking. The lung digest was filtered by a 40 mm sieve. After centrifuging, the cell pellet was resuspended in MACS (Miltenyi Biotec, Bergisch Gladbach, Germany) buffer. Subsequently, 10 mL of CD4 MicroBeads were added to per 10^7 total cells. After incubation for 15 min at 4 ℃, the cells were resuspended in MACS buffer. The cell suspension was applied onto the MACS column. After the sorting procedure, the magnetically-labeled $CD4^+$ cells were retained in the column. 1 mL of MACS buffer was pipetted onto the column. The magnetically-labeled $CD4^+$ cells were flushed out immediately and the $CD4^+$ cell suspension was stored on ice for further investigation.

HDAC6 activity.

HDAC6 activity measurement was performed as described previously [2]. Cell culture was lysed and analyzed using a fluorometric HDAC6 activity assay kit (BioVision, Milpitas, CA, USA). Cell viability of naive $CD4^+$ T cells was determined by trypan blue staining, as previously described [3].

Transfection and dual-luciferase assay

The binary Gal4 reporter plasmids (HIF-1a-N-TAD; HIF-1a-C-TAD) were generated as previously described [4]. Naive $CD4^+$ T cells were treated with (10 μM) or without Tubastatin A under Th17-skewing or normal conditions 24 h after the transfection to measure the effect of HDAC6i on HIF-TAD activity. Cells were harvested on day 6 after transfection. Dual-Luciferase Reporter Assay System (Promega, Madison, Wi, USA) was used for the measurement of luciferase activities.

References

1. Lin X, Li W, Lai J, Okazaki M, Sugimoto S, Yamamoto S, et al. Five-year update on the mouse model of orthotopic lung transplantation: Scientific uses, tricks of the trade, and tips for success. J Thorac Dis. 2012; 4: 247-58.
2. Zeng L, Xiao Q, Margariti A, Zhang Z, Zampetaki A, Patel S et al. HDAC3 is crucial in shear- and

VEGF-induced stem cell differentiation toward endothelial cells. J Cell Biol 2006;174(7):1059-1069.
3. Lee JH, Mahendran A, Yao Y, Ngo L, Venta-Perez G, Choy ML et al. Development of a histone deacetylase 6 inhibitor and its biological effects. Proc Natl Acad Sci U S A 2013;110(39):15704-15709.
4. Schoepflin ZR, Shapiro IM, Risbud MV. Class I and IIa HDACs Mediate HIF-1alpha Stability Through PHD2-Dependent Mechanism, While HDAC6, a Class IIb Member, Promotes HIF-1alpha Transcriptional Activity in Nucleus Pulposus Cells of the Intervertebral Disc. J Bone Miner Res 2016;31(6):1287-1299.

Supplementary Figure

Figure S1

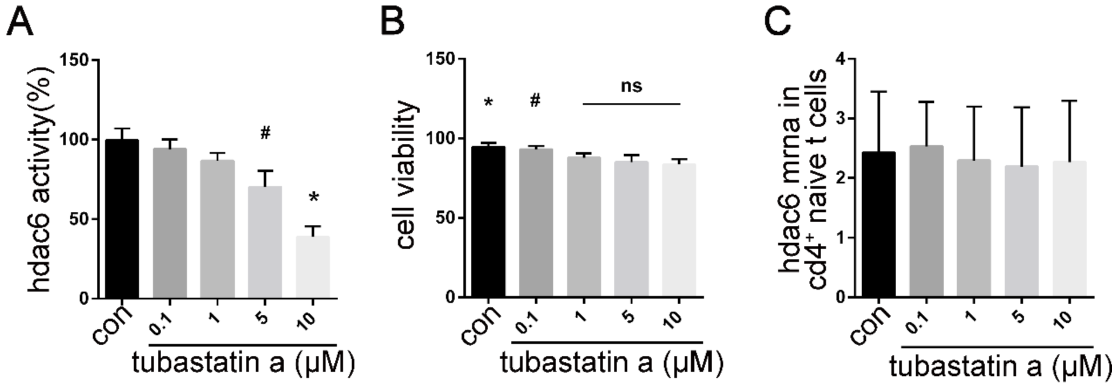

Figure S1. Effects of HDAC6 inhibitor Tubastatin A on HDAC6 activity and cell viability. After pretreating naive $CD4^+$ T cells with Tubastatin A (0.1, 1, 5, or 10 μM) or vehicle (DMSO) for 72 h, HDAC6 activity was measured by fluorometric HDAC6 activity assay. #: Tubastatin A (5 μM) vs. control and Tubastatin A (0.1, 1, and 10 μM), $p<0.05$; *: Tubastatin A (10 μM) vs. control and Tubastatin A (0.1, 1, and 5 μM), $p<0.05$ (A) Following 72 h of naive $CD4^+$ T cells incubation with Tubastatin A (0.1, 1, 5, or 10 μM) or vehicle (DMSO), cell viability was evaluated by trypan blue staining. #: Tubastatin A (0.1 μM) vs. Tubastatin A (5 and 10 μM), $p<0.05$; *: Control vs. Tubastatin A (5 and 10 μM), $p<0.05$ (B) and HDAC6 mRNA expression was measured by quantitative real-time PCR (qRT-PCR). The results were normalized to the glyceraldehyde-3-phosphate dehydrogenase (GAPDH) levels (C) con: naive $CD4^+$ T cells with vehicle treatment. All drug dilutions were done in cell culture media for a final DMSO concentration of 0.1% (vol/vol). Data are expressed as mean ± standard deviation. Data represent 3 independent experiments.

Figure S2

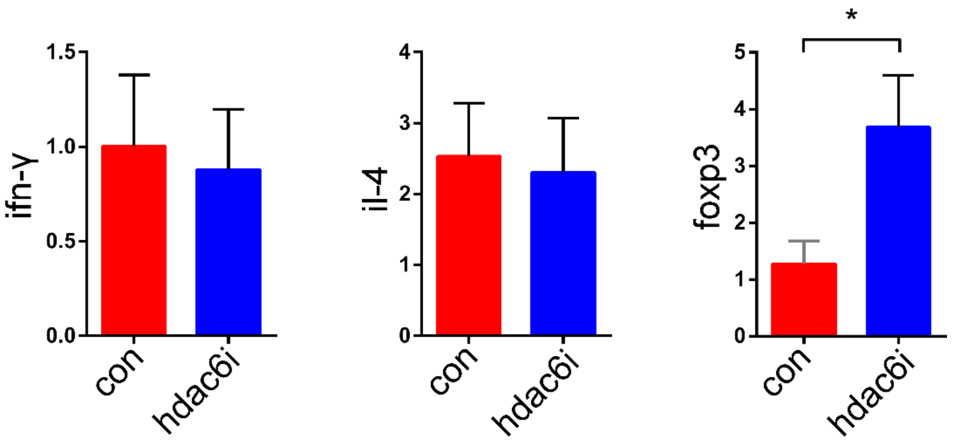

Figure S2. Effects of HDAC6i Tubastatin A on the expression of IFN-γ, IL-4, and Foxp3 mRNAs in naïve CD4$^+$ T cells under Th17-skewing conditions. mRNA expression levels of IFN-γ, IL-4, and Foxp3 in naïve CD4$^+$ T cells under Th17-skewing conditions with or without Tubastatin A treatment were measured by qRT-PCR. The results were normalized to the GAPDH levels. Data are expressed as mean ± standard deviation and represent 3 independent experiments, *: $p<0.05$.

Figure S3

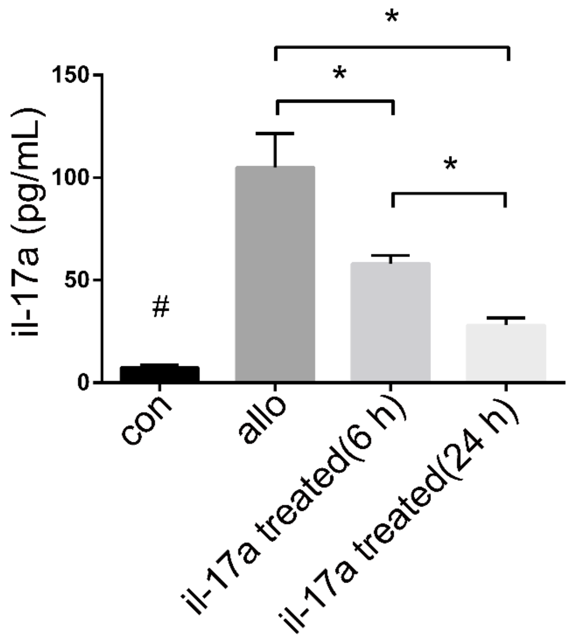

Figure S3. The IL-17A levels in sera of IL-17A-treated mice and lung allograft recipients. The sera of vehicle-treated mice, lung allograft recipients on POD 5, and IL-17A-treated mice (300 ng each mouse, iv) were used for testing the cytokine levels of IL-17A by cytometric bead array detection. In the IL-17A-treated group, sera were collected from mice at 6 h and 24 h after IL-17A treatment. In control group, IL-17A treatment group (6 h), and IL-17A treatment group (24 h), n=3; In lung allograft recipients group, n=5.

Figure S4

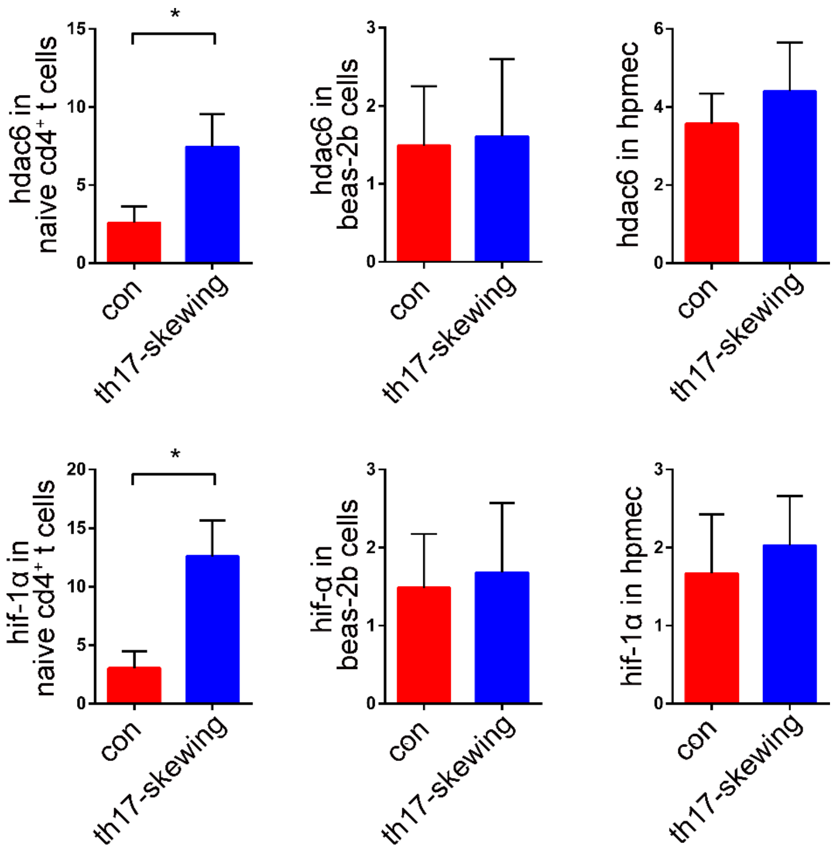

Figure S4: The mRNA expression of HDAC6 and HIF-1α in naïve CD4$^+$ T cells, BEAS-2B cells, and HPMECs under Th17-skewing conditions. The naïve CD4$^+$ T cells, human bronchial epithelial (BEAS-2B) cells, and human pulmonary microvascular endothelial cells (HPMECs) were cultured in normal conditions and Th17-skewing conditions for 5 d. The HDAC6 and HIF-1α mRNA were detected in these cell cultures by qRT-PCR. The results were normalized to the GAPDH levels. Data are expressed as mean ± standard deviation, Data represent 3 independent experiments, *: $p<0.05$.
